# Supplementary material for: Touch or click friendly: Towards adaptive user interfaces for complex applications
Source: PLoS One. 2024 Feb 5;19(2):e0297056. doi: 10.1371/journal.pone.0297056 (PMC10843409; doi:10.1371/journal.pone.0297056)
Supplement: S1 Appendix — (DOCX) [file pone.0297056.s001.docx]

**Appendix A1: Tasks list:**

The difficulty is formed on the total number of actions in a task. In some tasks, users were instructed to repeat the task and that repetition is considered an action.

| **S.**  **NO** | **TASKS descriptions** | **Difficulty Index** |
| --- | --- | --- |
| T1 | Start Microsoft word 2010. | 3 |
| T2 | Save the document.  On desktop.  Word Document format.  With default name which may be doc1. | 5 |
| T3 | Open document named “WELCOME COMSATS” location at desktop. | 4 |
| T4 | On document WELCOME COMSATS.  Set margin to Moderate.  Orientation to portrait.  Size to legal.  Column one. | 9 |
| T5 | 1. Set hyphenation to automatic.(right side of column option).  2 .Activate trackchanges option.  Show/hide review pane.  In Final show markup option set original.  Deactivate trackchanges. | 9 |
| T6 | Select ,select All document (on ribbon rightmost option)  Change font to “Times New Roman”.  Font size to 12.  Select 3rd paragraph.  Italicize(ITALIC) the 3rd paragraph.  Change case to upper case.  Use strikethrough option.  Highlight with yellow color. | 14 |
| T7 | Apply undo 3 times and then single redo option. | 4 |
| T8 | Cut the “Established/charter” heading with data and paste it under the “vision” heading paragraph . | 4 |
| T9 | Change the font color of word “Established/charter” to red.  Through apply format painter change color of word “mission to red.  Apply Clear formatting on word “Established/charter” | 7 |
| T10 | Apply text effect option on word “Historical Perspective”.  Change style to heading 1. | 4 |
| T11 | On title “CIIT’s History and Quick Facts”.  Use wordart (fill- tan text 2- outline background 2) which is first in list | 4 |
| T12 | Select first two paragraphs.  Align justified  1.5 line spacing  increase indent two clicks (8 points) | 7 |
| T13 | in data of “Established/charter” heading.  In 1998 make 8 to SUPERSCRIPT and  In 2000 make last 0 SUBSCRIPT. | 4 |
| T14 | Convert to text the table1.  Apply bullets on text which is just converted from table1.  Sort these bulleted data in descending order. | 9 |
| T15 | Make quick parts of the bulleted data (table that is convert to text)  Use this quick part just under the original bulleted list. | 7 |
| T16 | after the quick part  Insert Object of bitmap type( write A in paint working area)  Insert date just after the last paragraph which is ended with word govt and community group. | 11 |
| T17 | Insert symbol any e.g.@ after date and time inserted in last paragraph. | 3 |
| T18 | Insert an equation any e.g. area of circle after symbol inserted in last paragraph. | 3 |
| T19 | Select last paragraph.  Apply paragraph border of type box  and 3-D page border on all pages  apply red paragraph shading. | 11 |
| T20 | Set water mark of type “Don’t copy”  and page color of light green from standard colors. | 5 |
| T21 | Change the document to draft view than print layout view,  show/hide ruler,  Set Zoom to page width  than set zoom to 105  and show/hide navigation pane . | 15 |
| T22 | Page break after bitmap image A,  and insert blank page after breaked page  and Insert cover page of type ( Alphabet) as first page. | 6 |
| T23 | Insert table of contents of type automatic table 2 on new blank page just inserted.  by changing style of “established/charter” word to heading 1, “vision” to heading 2 and “mission” to heading 2, update table of contents. | 15 |
| T24 | Insert hyperlink of word “vision” with any word document present on desktop. | 6 |
| T25 | Insert header of type blank and  insert page numbers on ( top of the page of type plain number). | 4 |
| T26 | Beside word “distinction” which is highlighted with yellow color.  Insert cross reference of (type heading with the heading of “historical perspective”). | 8 |
| T27 | First paragraph first word “the” and first alphabet T.Apply drop cap type (droped ). | 3 |
| T28 | Record a macro on 3rd paragraph (bold, italic and underline) and run macro through a button (smiley) from the quick access toolbar on 4th paragraph. | 20 |
| T29 | At the end of 2nd paragraph  Insert a shape rectangle  Fill color of type red in shape.  2. rotate the shape to 90 degree right. | 9 |
| T30 | Set the position of the shape at top right corner and use Wrap text of type behind text . | 5 |
| T31 | Insert Caption named figure 1 and  create table of figures under the table of contents. | 7 |
| T32 | By marking five words one by one (Campuses,Faculties,Academic department,Research centre,students) Insert index after table of figures.  and hide the paragraph marker. | 21 |
| T33 | Mark citation of two words “ COMSATS” and “TWAS” one by one in first paragraph to Insert table of authority after index.  Hide the paragraph marker. | 13 |
| T34 | 1.Create New blank document and  2.view document side by side both the newly created and doc1. | 8 |
| T35 | On New document Add a picture e.g. tulip resize the picture(not using ribbon) into less than 4x4 size.  Apply picture border of type weight 6pt  and picture effect of type glow variations. | 11 |
| T36 | Crop the picture in oval shape. | 4 |
| T37 | Insert smart art of type process named continuous arrow process.  Resize it by less than 4x4 size. | 6 |
| T38 | 1.Add a clipart e.g. business man and  Add a chart type Pie.  2.Show/hide selection pane. | 12 |
| T39 | Add a screen shot of ribbon of microsoft word 2010. | 4 |
| T40 | 1.Save–as this document with default name like doc2 at desktop and  2.switch windows to doc1 which is blank. | 7 |
| T41 | On doc 1 Insert table of 5x5.with insert table window by adjusting rows and columns. | 7 |
| T42 | Move a table to few lines downward direction and resize it . | 5 |
| T43 | Add a row at the end and add a column at rightmost of the table | 5 |
| T44 | In cell margin set margin to 0.05 top and bottom. Check button of Allow spacing between cells and  set text direction downward. | 5 |
| T45 | 1.Merge cells of first two rows and  align top centre and  2.apply yellow shading option. | 6 |
| T46 | Split cells which was merged into two rows and two columns. | 4 |
| T47 | Erase single line of top right cell of the table.  Format a table by giving table grid style. | 6 |
| T48 | Delete last row,  Delete rightmost column  Delete table. | 9 |
| T49 | Close the document doc1 with don’t save option. | 3 |
| T50 | Word Count in the document WEL COME COMSATS. language set English US as a default language. | 6 |
| T51 | Apply spelling &grammar check on WELCOME COMSATS document and replace the wrong word if any with correct word atleast two words. | 3 |
| T52 | Change with synonyms the word aim which is at 2nd line of first paragraph (THESAURUS) . | 4 |
| T53 | Change the theme of the document to angles and add line numbers of type continuous. | 5 |
| T54 | See the print preview of all the pages of document | 4 |
| T55 | Mark as final the document “WELCOME COMSATS”.(protect document) | 5 |
| T56 | Exit out from Microsoft Word. | 3 |
|  |  |  |
