# Supplementary material for: Touch or click friendly: Towards adaptive user interfaces for complex applications
Source: PLoS One. 2024 Feb 5;19(2):e0297056. doi: 10.1371/journal.pone.0297056 (PMC10843409; doi:10.1371/journal.pone.0297056)
Supplement: S2 Appendix — (DOCX) [file pone.0297056.s002.docx]

**Appendix A2: Detailed Tasks List**

| **Task No** | **Steps to perform tasks** |
| --- | --- |
| **T1** | 1. Click Start button.   The Start menu appears. You might find the Microsoft Word 365 program icon right there on the Start menu. If you see the Word icon,   1. Click Microsoft Word 365 to run the program.   If word 365 is not here than go to Step 4.   1. Click All Programs.   All Programs changes to Back once you click it.   1. Click Microsoft Office. 2. Click Microsoft Word 365.   A blank document appears.  If using window 8  7. Click on start button.  8. Scroll the screen upward  9. Click on microsoft word 365 |
| **T2** | Before you save a document, Word displays a generic name in the title bar.  1. Click the File tab.  The Backstage view appears.  2. Click Save.  The Save dialog box appears.  3. Word automatically selects the first line or first several words of your document as a filename and puts it in the Save dialog box. If that’s okay, you can move to Step 4. Otherwise, type a name in the File Name box.  4. Word save document to default location. You can browse to select new locations or create new folder by clicking new folder option to save the document.  5. If you want to change type of document e.g. PDF format click on Save as type option select desired type.  6. Click Save.  Word saves the document and displays the name you supplied in the title bar. |
| **T3** | 1. Click the File tab.   The Backstage view appears.   1. Click Open.   The Open dialog box appears.   - 1. Click here to navigate to the folder containing the document you want to open.Documents in a folder appear here.   2. Click the document you want to open.   3. Click Open. |
| **T4** | 1. Click anywhere in the document or section where you want to change margins.  2. Click the Page Layout tab.  3. Click Margins.  The Margins Gallery appears. If the margins you want to use appear in the Margins Gallery,  4. Click your desired margin.  and skip the rest of these steps; otherwise, proceed with Steps 5 to 9.  5. Click custom margin.  6. Drag the mouse pointer over any margin setting.  7. Navigate a new margin setting.  8. Repeat Steps 5 and 6 for each margin setting.  9. Click ok. here if you want to change the paper setting navigate and set your paper size and click ok.  10. In page setup window click orientation from ribbon.  11. Select portrait or landscape.  If you want to make columns of your document.  12. Click on column option.  13. Select your desired number of column. |
| **T5** | 1. Click the Page Layout tab .  2. Click the Hyphenation button near column and water mark option.  3. Click on hyphenation option. OR direct click on automatic option.  Dialogue box appears  4. Check the automatic hyphenate option.  5. Click Ok.   1. Click the Review tab. 2. Click the Track Changes command. It should now be highlighted in gold to show that it is active. Any changes you make to the document will be shown as colored markups. 3. Click the Track Changes command again to turn it off. 4. When track is changed of few text we can see original text without change or final document by clicking on final show markup button. 5. We can see reviw pane vertical or horizonta by clicking on review pane button.and click again to remove the review pane. 6. Changes made can be selected or rejected by cicking on select and reject button. |
| **T6** | To select the complete document  1- Click on Home tab.  2- at right corner of the ribbon click on select.  3-Click select all.  3- Click on Font option to display a list of the available fonts on your computer.  Word displays a sample of the selected text in any font at which you point the mouse.  4-Click the font you want to use.  Word assigns the font you selected to the text you selected.  Click anywhere outside the selection to continue working.  5- Click on small arrow beside the font size option. a list of possible sized of current font appears.  6-Click on desired font size word assigns the font size you selected to the text.  If you have font 12 or less and you want to increase or decrease font size by 1  7- You can do this by simply click on Grow font or shrink font option front of font size option.  Click anywhere outside the selection to continue working.  8-Select your desired text whom you want to change into bold , italic etc.  8- Click Bold/Italic/Underline button. If underline option is selected and you want to change the style of line.  8.1 Click on small arrow near underline option.  8.2 Click your desired line  if you want to give colors to line than  8.3 Click on underline color  8.4 Click your desired color.  8.5 Click on more line  8.6 Select your desired line.  Word applies the emphasis you selected.  Click anywhere outside the selection to continue working.  9- Click on change case option just under the maiing tab.  10- Click on your desired case like uppercase or toggle case.  Selected text appears in new case.  Click anywhere outside the selection to continue working.  11.click on text highlight option under the change case option.  12. click on your desired color.  13- Click on strikethrough option just under the font size option.  Word applies the emphasis you selected. |
| **T7** | If you mistakenly delete text or any other action you want to revert,Than you use the Undo feature, you can recover the text.   1. Click the Undo button in Quick access toolbar in upper-left corner of the ribbon.   Word reverses the effects of the last change you made.  You can repeatedly click to reverse each action you have taken, from last to first.  2- If you decide not to reverse an action after clicking, click the Redo button. |
| **T8** | 1. Select the text you want to cut or copy.  2. Click the Home tab.  3. Click the Cut button to cut text, click the Copy button at left corner of the ribbon.  4. Click to place the insertion point at the location where you want the text to appear.  5. Click the Paste button or down arrow under paste button  Buttons representing paste options appear.  5.1 To preview the appearance of the selection, point at the Keep Source Formatting button .  5.2 To preview the appearance of the selection, point at the Use Destination Styles button.  5.3 To preview the appearance of the selection, point at the Keep Text Only button.  the text appears at the new location.  6. Select your desired option. |
| **T9** | 1. Select the text that you want to change to a different color.  2. Click on Home tab.  3. Click the Font Color button( right side of text highlight )on the Ribbon and point at a color. Word displays a color sample of the selected text.  4. Click on any color from theme colors or standard colors.  If you want to try other colors  4.1 Click on more colors.  A new window named colors appears.  For standard colors  4.1.1 Select color from standard colors.  4.1.2 Press ok.  If you don’t selected the standard color and want to select custom color.  4.2 Click on custom.  4.2.1 Select color model.  4.2.2 Select combination of Red, green and blue colors.  4.2.3 Click on Ok.  4.3 Click on gradient if you don’t select more color options  4.3.1 Click on your desired color.  If you want to try other color.  4.3.2. Click on more gradients.  A new window opens of Format text appears  4.3.3 Adjust your desired option.  4.3.4 Click on close.  For applying format painter  5. Select the text or graphic that has the formatting that you want to copy. Note: If you want to copy text formatting, select a portion of a paragraph.  6. Click on format painter button under cut copy option .  7.Use the brush to paint over a selection of text or graphics to apply the formatting. ...  If you want use clear formatting option.  8.select the text whom formatting you want to clear.  9. Click on clear formatting button under mailing tab on ribbon.  Formatting will be clear. |
| **T10** | 1. Select the text to which you want to apply an effect.  2. Click the Home tab.  3 Click the Text Effects button.  The Text Effects gallery appears.  Word displays a sample of the selected text in any text effect at which you point the mouse.  4. Click an option from the gallery to apply it.  If you want to select text effect from other options move the mouse pointer on outline or shadow or reflection or glow option.  5. Click on your desired effect.  6. Select one example of text the formatting that you want to change.  7. Click the Home tab.  8. Click the Styles. Your desired directly heading 1 or heading 2 etc.  Or  9. Position the mouse pointer over a style until appears.  10. Click to display a list of options.  11. Click Select All Instance(s).  Word selects all text in your document formatted using the style of the text you selected in Step 1  12 Click the style you want to apply to all selected text.  Word changes all selected text to the style you selected in Step 7.You can click anywhere to continue working. |
| **T11** | 1. Select existing text.  2. Click the Insert tab.  3. Click WordArt right side of the ribbon.  The WordArt Gallery appears.  4. Click the WordArt style you want to apply.  Your text appears selected in the WordArt style you applied;  5. Click anywhere to continue working. |
| **T12** | 1. Click anywhere in the paragraph or select the paragraph or text that you want to align.  2. Click the Home tab.  3. Click an alignment button.  The Align Left button aligns text with the left margin, the Center button, centers text between the left and right margins, the Align Right button aligns text with the right margin, and the Justify button aligns text between the left and right margins. Word aligns the text.  4. Click the Line Spacing button right of justify option.  4.1 Click a number.  1 represents single spacing, the default in Word 97–2003; 1.15 is the default spacing in Word 365; 1.5 places 1⁄2 blank line between lines of text; 2 represents double spacing; 2.5 places 11⁄2 blank lines between lines of text; and 3 represents triple spacing.Word applies the line spacing you specified to the selected text.  If you want to increase or decrease the indent  5. Select your desired text.  5- Click on increase or decrease indent button.just under the developer tab. |
| **T13** | 1. Select the text that you want to make superscript or subscript.  2. Click the Home tab.  3. Click the Superscript button or the Subscript button.under the font size option.  Word superscripts or subscripts the selected text.  You can click anywhere outside the selection to continue working. |
| **T14** | 1. Select the table by clicking on right upper corner a handle.  When table is selected on ribbon two more tabs design and layout appears.  2. click on layout tab.  3. Click on covert to text option which is second last option on right side of the ribbon.  Convert table to text diaglogue box appears  4. click on ok.  1- Select the text to which you want to assign bullets or numbers.  2- Click the Home tab.  3- Click the bullets button. Just under the reviw tab.  Word applies bullets to the selection.  If you want to select Numbering  click on numbering button.  3.1Word applies the number to the selection.  3.2 Click anywhere outside the selection to continue working.  If you want to use multilevel number list  4- Click on multilevel list.  5- Select your desired list.  If you want to sort the bulleted or numbered text or digits  6.Select the text in a bulleted or numbered list.  7. select your desired text whom you want to sort.  8.click on the Sort under the developer tab .  Sort text window appear  9. click Paragraphs and Text, and  9. then click either Ascending or Descending  10. click Ok. |
| **T15** | 1. Select the text you are using frequenty.  2. Click on insert tab.  3. Click on quick parts.  4. Click on save selection to quick part gallery.  A dialogue box appear where default name will appear .  5. click ok.  Your desired selected text is saved.  6. Place the cursor where you want that text appears  7. Click on insert tab.  8. Click on quick parts.  Your saved text appears here .  9. Click on the text.  It will appear whenever you want as quick text. |
| **T16** | 1. Place the cursor where you want the date is appear.  2. Click on insert tab.  3. Click on date and time button.(right end of ribbon near equation option)  A dialogue box of different formats of date and time is appear.  4. Click on your desired format.  5. Click on Ok.  To insert object  6. place the cursor where you want to object will appear.  7.Click on Object.Near the equation option.  Downward menu appears here you can select object and text from file.  8. Here we select object. Object window will appear.here you can select different types of object files  9.Here we select bitmap image.  10. Click on Ok.  Microsoft Paint window will appear.  10. at working area you can write something as default no selection is required.  11. close the paint window  Object will appear at your desired location |
| **T17** | 1. Click the location in the document where you want the symbol to appear.  2. Click the **Insert** tab.  3. Click **Symbol**.  A list of commonly used symbols appears. If the symbol you need appears in the list,  4. Click the symbol and skip the rest of these steps.  5. Click **More Symbols**.  The Symbol dialog box appears.  6. Click here and select the symbol’s font  The available symbols change to match the font you selected.  7. Click a symbol.  8. Click **Insert**.  9. Click **Close** to close the Symbol dialog box. |
| **T18** | **1**.Position the insertion point where you want to insert an equation.  2. Click the Insert tab.  3. Click on small arrow beside the Equation button.  The Equation Gallery, a list of commonly used equations, appears.  4. click an equation to insert  5.Click outside the equation box to continue.  and then skip Steps 6 and 7.otherwise  6. Click Insert New Equation.  Word inserts a blank equation box. The Equations Tools Design tab appears on the Ribbon.  7. Type your equation.You can click the tools on the Ribbon to help you type the equation.  8. Press or click outside the equation box.  Word hides the equation box and you can continue typing. |
| **T19** | 1. Select the text that you want to surround with a border.  2. Click the Home tab.  3. Click beside the Borders button under paragraph symbol.  Downward menu appears at last option.  4. Click Border and Shading.  The Borders and Shading dialog box appears.  5 Click the Borders tab.  6 Click to select a type of border like box, 3-D etc.  If you want to change the border line, color of the line and thickness of the line and page borders go to 8 else  7. Click Ok  8.Click to select the style for the border line.  8.1 Click to select a color for the border line.  8.2 Click to select a thickness for the border line.  You can see results of the settings if you want to apply page borders and shading go to 10 else.  9. Click Ok  The border appears around the text you selected in Step 1.  If you want to give border to whole page  10. Click on page border tab.  12. Select your border type box etc. If you want to change the features of border line and want to apply shading go to 14 else.  13. Click ok  14. Select style of border.  14.1 Select width of border.  14.3 Select color of page borders. Border you selected can be seen.  14.4. Select the option of single page or whole document for borders. If you want to apply shading go to 16 else  15. Click Ok  16 Click on shading tab.  17. Select your desired color in Fill for shading.  18. Click anywhere outside the selection to continue working |
| **T20** | 1. Click to display your document in Print Layout view by clicking on right of status bar on printlayout view.If it is already in printlayout view simple click the document ignore printlayout.  2. Click the Page Layout tab.  3. Click Watermark arrow symbol under review tab.  4. Select your desired water mark.  If you want to make watermark of picture or your on text than follow the following steps  5. Click Custom Watermark.  The Printed Watermark dialog box appears.  6. Select the Text watermark option .  7. Click here and select the text to use as a watermark or type your own text.  You can use these options to control the font, size, color, intensity, and layout of the watermark.  8. Click OK.  If you want to give page color than  9. Click on page color right side of watermark option.  Theme colors standard colors and other options appear.move the mouse pointer over a color of the page appears  10. Select your desired color.  Word displays the watermark on each page of your document |
| **T21** | 1. Click the View tab.  2. Click one of the Document Views buttons on the Ribbon:  Print Layout, Full Screen Reading, Web Layout, Outline, Draft  3. You can show or hide the ruler by checking and unchecking the ruler option.  4. Click Zoom  Zoom window appears.  5. Select your desired option by clicking or by navigation in percent option.  6. You can check the radio button of page width, one or two pages option.  7. You can check the gridines button to show grid lines.  8. You can check navigation pane button to show navigation pane  Navigation pane appears left side click again to remove it. |
| **T22** | 1. Position the insertion point immediately before the text that you want to appear on a new page.  2. Click the Insert tab.  3. Click Page Break.  Word inserts a page break and moves all text after the page break onto a new page.  For inserting blank page.  4.Click on Insert.  5.Click on blank page.  To insert coverpage click on top of the document.  Click on insert.  6. Click on cover page option.  Different built in cover appears  7. Click on your desired cover page. |
| **T23** | For inserting table of contents.  1. Place the insertion point in your document where you want the table of contents to appear.  This example places the table of contents on a blank page.  2. Click the References tab.  3. Click Table of Contents on ribbon leftmost option.  The Table of Contents gallery appears.  4. Click a table of contents layout.  Generate a Table of Contents  Word inserts a table of contents at the location of the insertion point. The information in the table of contents comes from text to which Heading styles 1, 2, and 3 are applied. You can continue working in your document, adding new text styled with heading styles.  When you adding more text in to your document and add heading and sub-heading then  5.Click on table of contents anywhere.  6. At the top of table of contents update table button appears click on it.  Dialogue box appear  7..Check complete table option  8.Press OK.  If you want to remove table of contents.  9. Click on Reference tab.  10. Click on table of contents.  11. Remove table of contents. |
| **T24** | Select the text or image you want to make a hyperlink.   1. Click the Insert tab. 2. Click Hyperlink option.   -The Insert Hyperlink dialog box will open.  4. Browse for your file whom with you want to make hyperlink.  5. Click Ok button. |
| **T25** | 1. Click the **Insert** tab.  2. Click **Header** or **footer**  The Header and Footer Gallery appears.  3. Click a Header style or footer style e.g. blank.  The text in your document appears dimmed. The insertion point appears in the Footer area. Header & Footer Tools appear on the Ribbon. You can move to header or footer by clicking header and footer button.. Header or footer will appear in your document.You can move between header and footer by clicking go to header or go to footer button  4. double click on under the header area to make normal the dimmed text and save the header.  For closing header and footer ribbon.  5. Click **Close Header and Footer red cross symbol in the ribbon.**  For inserting page numbers  5. Click Page Number.  Page number placement options appear.  6. Click a placement option.  A gallery of page number alignment and formatting options appears.  7. Click your desired option.  The page number appears in the header or footer. |
| **T26** | 1. Select the text whose cross reference you want to create. For example, "See Figure 2on page 1on page 1 for an explanation of the upward trend." 2. Click On the **Insert** tab, 3. Click **Cross-reference**. 4. In the **Reference type** box, click the drop-down list to pick what you want to link to. The list of what's available depends on the type of item (heading, page number, etc.) you're linking to. 5. In the **Insert reference to** box, click the information you want inserted in the document. Choices depend 6. In the **For which** box, click the specific item you want to refer to, such as "Insert the cross-reference." 7. To allow users to jump to the referenced item, select the **Insert as hyperlink** check box. 8. If the **Include above/below** check box is available, check it to include specify the relative position of the referenced item. 9. Click **Insert**. |
| **T27** | 1. Select the letter you want to insert as drop cap.  2. Click on the "Insert" tab,  3. Click on "Drop Cap" button.  hold the mouse pointer over your choice to see a preview in your document.  4. Click on your desired drop cap option.  Dropcap of your desired alphabet appears. |
| **T28** | 1. Select the text on which fomating macro you want to record  2.Click the View tab.  3. Click the arrow under Macros.  4. Click Record Macro.  The Record Macro dialog box appears.  5. Type a name for the macro.Or leave the by default name.  *Note: Macro names must begin with a letter and contain no spaces.*  6. assign the macro buton or keyboard shortcut here. Here we clcik button  A dialogue box appear here  7. click on the name of your macro here  8. Click on add button macro will be add in quick access toolbar.  If you want to give a shape for button here after add  9. click on modify button  A dialogue box appears here with different shapes  10. Click on desired shape for button here  11. click on ok  12. Press ok of main dialogue box  The mouse pointer changes.Perform the actions you want included in the macro.*Note: Macros can include typing, formatting, and commands. You cannot use the mouse to position* *the insertion point.*  now you can bold italic underline the text or any formatting you want will be recorded.then  13. When you have taken all the actions you want to include in the macro, click under Macros.  14. Click Stop Recording.  Word saves the macro.  15. Position the insertion point in your document where you want the results of the macro to appear.or select the text on which you want to run macro.  16. Click the View tab and apply 16 onward stepes OR click on the button appears on quick access toolbar.  17. Click Macros.  The Macros dialog box appears.Available macros appear here.  18. Click the macro you want to run.  The macro’s description appears here.  19. Click Run.  Word performs the actions stored in the macro. |
| **T29** | 1.Click the **Insert** tab.  2. Click **Shapes**.  The Shapes Gallery appears.  3 Click a shape.  4. Position the mouse pointer at the upper-left corner of the place where you want the shape to appear.  5. Drag the mouse pointer down and to the right until the shape is the size you want  When you release the mouse button, the shape appears. Drawing tools also appears at the ribbon.  6.click on design tab.  Click on fill color.  7. Select your desired color.  8. Click on shape outline.  9. Select your desired color for outline the shape.  10. click on rotate.  11. Click on your desired rotation. |
| **T30** | You can select the position of your shape where you want to place the shape  1. Click on shape.  2.Click on format tab.  3.Click on position..  4. Select your desired position.    1. Click a graphic (shape or picture).  Handles appear around the image.  2. Click **Format tab** on the ribbon.  3. Click **Wrap Text**.  4. Click the wrapping style you want to apply.  Word wraps text around the graphic using the text wrapping option you selected.    7.Click on align  Select your desired alignment for graphic. |
| **T31** | 1. Select the object (table, equation, figure, or another object) that you want to add a caption to. 2. Click On the References tab. 3. In the Captions group, Click Insert Caption.   4.In the Label list, select the label that best describes the object, such as a figure or equation.  If you insert few caption to some graphic than  5. Click where you want to insert the table of figures.  6. Click on References tab,  7. In the Captions group, click Insert Table of Figures.  8. Click Options.  9. Select the Style check box.  In the Style list, click the style that you applied to the captions, and then  10. Click OK |
| **T32** | 1. Select the words or phrases you want to enter in index.  2. Click on Reference tab.  3. In index section of ribbon click on mark entry button.  A dialogue box will appear  4. Click on mark  5. Click cancel  Mark few words you want to insert in index with same process.  6. Click on place or take the cursor to place where you want to insert index.  8. Click on insert index.  Index will be created there  With index paragraph symbol may appear to remove these symbols  9. click on Home tab.  10. click on paragraph symbol under developer tab right of sort option. |
| **T33** | 1. Select the words or text.  2. Click on Reference tab.  3. Click on mark citation.  A dialogue box appears  4. Click on mark  5. Click on category to select desired category cases or rules etc.  5. Click cancel. Repeat these steps to mark citations more than once  6. Click where you want to insert table of authorities.  7. Click insert table of authorities. A dialogue box will appear.  8. Click on your desired authority.  9. Click Ok.  10. Click the home tab.  11. Click the paragraph marker button. |
| **T34** | 1. Click the **File** tab.   The Backstage view appears.   1. Click **New**.   Templates available on your computer appear here.   1. Click an available type of template etc. blank document. 2. Select a template to use for your document. OR click on blank document   A preview of the template appears here.   1. Click **Create**.   The new document appears. You can edit this document any way you choose.  6- Click on View tab.  7-Click on view side by side.A compare window will appear.which shows the documents  8-Click Ok .You can see documents side by side. |
| **T35** | 1. Click in your document where you want to add a picture.  2. Click the Insert tab.  3. Click Picture.  The Insert Picture dialog box appears.  The folder you are viewing appears here.  You can click here to navigate to commonly used locations where pictures may be stored.  4. Navigate to the folder containing the picture you want to add.  5. Click the picture you want to add to your document.  6. Click Insert.  Picture will appear in your desired locaiton.  7.click on picture on ribbon format tab appears  8. you can change picture border by clicking on picture border option  9. select your desired border color.  10. layout of picture can be changed by clicking on layout picture.  11. you can change picture effect by clicking on picture effect.  12. to resize the picture click on picture.  13. handles appears click on handles and drag upward downward right left to resize the pic.  Size of the picture appears on the ribbon. |
| **T36** | 1. Click the image to select it; handles ( and ) surround the image.  2. Click on Format tab after developer tab on the ribbon.  3. Click Crop.OR click on crop to shape.  . If click on Crop  Black crop handles appear at each corner and in the middle of each side of the image, framing the portion of the image that will remain.  4. Slide the mouse pointer over a crop handle ( , , or changes to ,, , , , , , or ).Crop a Picture  5. Click and drag the handle toward the center of the picture.  As you drag, the crop handle changes to .Word displays the area it will remove shaded in black.  6. Repeat Steps 4 and 5 as needed until crop handles frame the portion of the image you want to keep.  Areas to be removed appear shaded in black.  7. Press The cropped image appears.  You can press or click outside the image to cancel its selection.  .  OR if click on crop to shape. Ignore steps 4,5,6,7  A dialogue box appears of different shapes  8. Click your desired shape on which you desired to crop your picture. |
| **T37** | 1- Place the cursor where you want to enter the smart art.  2- Click on Insert Tab.  3- Click on smart Art.  Smart art graphics window appear  4- Select your desired smart art by scrolling down.  5- Click on Ok.  Smart art will appear at your desired location.  You can resize it by dragging through handles |
| **T38** | 1. Click the location in the document where you want to place the clip art. 2. Go to the "Insert" tab. 3. Select the "Clip Art" option. ...   Search your clip art for an image for what you have in mind.right side of the window Clipart window appear  4. click on GO button.  5.Different clipart appears scroll down to select your desisred one.  6.Click on desired clipart.  It wil appear on your desired location.  7. cancel the clipart window  Click anywhere to continue working.  8. click the Chart command in the Illustrations group. ...  A dialog box will appear. ...  9. Select the desired chart,  10. click OK. ...  A chart and a spreadsheet will appear. ...  11. Cross the spread sheet  12. Click anywhere to continue working.  13. Click on Page layout.  14. Click Selection Pane.once again click on it to hide this.  The Selection and Visibility Pane appears. A list of graphic objects on the current page appear. Go to your We can hide and show all the objects here.you can hide single objects or more manually by clicking hide button |
| **T39** | 1.Open the Word document in which you want to insert a screenshot if opened already ignore this.  2. Position the insertion point where you want the screenshot to appear  3 Click Insert  4.Click Screenshot.  The Screenshot Gallery shows open programs and available screenshots of those programs. Click the screenshot you want to insert in your Word document. The screenshot appears selected in your Word document.  4.1 Click on screen clipping.  Word screen will be dimmed  4.2 drag over the position whom you want to take snapshot.  Just release the drag screenshot will appear on your document.  5.Click anywhere outside the screenshot to continue working. |
| **T40** | if you want to save a document with some other name and location.  1. Click the File tab.  Backstage view appears.  2. Click Save as.  Dialog box appears    3. Word automatically selects the first line or first several words of your document as a filename and puts it in the Save dialog box. If that’s okay, you can move to Step 4. Otherwise, type a name in the File Name box.  4. Word save documents to default location you can select new locations.(if not change skip this step)  5. If you want to change document type select your desired type by clicking save as option.(if not change skip it)  6. Click Save. Word Save the document in other location.  6.Click on view tab.  7.Click on switch document right side of the ribbon.  8.Select your desired document whom which you want to switch. |
| **T41** | 1. Click in your document where you want the table to appear.  2. Click the Insert tab.  3. Click Table.  Word displays a table grid.  4. Slide the mouse pointer across the squares that represent the number of rows and columns you want in your table. OR Click on insert table. Live Preview draws a sample of the table on-screen. OR dialogue box of create table appears.  5. Click the square representing the lower-right corner of your table. OR navigate to select number of rows and columns.  6. Press Ok.  The table appears in your document. Table Tools appear on the Ribbon. |
| **T42** | 1. Click on print layout view in bottom right side. If it is already in printlayout ignore this step.  2. Position the mouse pointer over the table.  A handle + like symbol appears in the upper-left corner of the table and small handle sign lower right corner.  3. Position the mouse pointer over the handle upper left corner.  4. Drag the table to a new location.  A dashed line represents the proposed table position.  5. Release the dragging.  The table appears in the new location.  6. Position the mouse pointer over the handle lower right corner.  7. Drag the table up to make it shorter or down to make it larger changes to .  *Note: You can also drag diagonally to simultaneously change both the width and height of the table.*  A dashed line represents the proposed table size.  8. Release the dragging to change the table’s size. |
| **T43** | 1. Click in the row below where you want a new row to appear.  2. Click the Layout tab.  3. Click Insert below /Above.  Word inserts a row and selects it. You can click in the row to add information to the table.  4.Click in the column to the right/left of the column you want to add new column.  5. Click Insert Right/left.  Word inserts a new column in the table to the right of the column you clicked in Step 1 |
| **T44** | 1. Click anywhere in the table.  2 Click the Layout tab.  3. Click Cell Margins.  The Table Options dialog box appears.  4. set desired margin settings here.  5. Select the Allow spacing between cells option and type a setting for space between cells.  6. Click OK.  7. Click on text direction to select your desired direction by clicking this icon. |
| **T45** | 1. Position the mouse pointer inside the first cell you want to merge.  2. Drag across the cells you want to merge to select them.  3. Click the **Layout** tab.  4. Click **Merge Cells**.  Word combines the cells into one cell and selects that cell.  For a table title, you can  5. click the **Align Center** button to center text in the cell both horizontally and vertically.  6. Click anywhere to cancel the selection.  7. Click design tab.  8. click shading.  Different color option appear.  9.click on desired color. |
| **T46** | 1.Click anywhere in the cell you want to split.  2. Click the Layout tab.  3. Click Split Cells.  The Split Cells dialog box appears.  4. Type the number of columns and rows into which you want to split the cell here. Or navigate the number of columns and rows.  5. Click OK |
| **T47** | 1.Click anywhere in the table.  2. Click on design tab.  3.Click on eraser which is rightmost option on ribbon  4. Apply selected raser on any line the line will be erased.  5. Click on eraser again to deactivate the eraser.  6. Position the mouse pointer over a table style.  Live Preview displays the table in the proposed table style.  6. Click the table style you want to use.  Word displays the table in the style you selected. |
| **T48** | 1. Click anywhere in the table you want to delete.  2. Click the Layout tab.  3. Click Delete.  4. Click Delete Table, column or row.  Word removes the table and its contents from your document. |
| **T49** | 1. Click the **File** tab.   The Backstage view appears.   1. Click **Close.**   Word removes the document from your screen. If you haven’t saved your document recently, Word prompts you to save before you close. If you had other documents open, Word displays the last document you used; otherwise, you see a blank Word window.  If you not save the document.  3- Click on save or not save save button. |
| **T50** | 1. Right-click the status bar.  The Status Bar Configuration menu appears. The number across from Word Count is the number of words in the document.  2. If no check mark appears beside  Word Count, click Word Count; otherwise, skip this step.  3. Click anywhere outside the menu.  OR  1. Select your text or document.  2. Click on review tab.  3. Click on word count option.  Word count window will appear having information about words pages etc.  4. Click on close to continue.  5.Click on language.  6.Click on set proofing language.  7.Click on set default language.  8.A dialogue box appear  9.Clcik on yes  10.click on ok. |
| **T51** | 1. Click the Review tab.  2. Click Spelling and Grammar.  Word selects the first spelling or grammar mistake and displays the Spelling and Grammar window.  *Note: If your document contains no errors, this window does not appear.*  Upper area displays the spelling or grammar mistake.  Lower area displays suggestions to correct the error.  3. Click the suggestion you want to use.  4 Click Change.  You can click Ignore Once or Ignore All to leave the selected word or phrase unchanged. Word selects the next spelling or grammar mistake.  5. Repeat Steps 3 and 4 for each spelling or grammar mistake.  Word displays a dialog box when it finishes checking for spelling and grammar mistakes.  6 Click OK. |
| **T52** | 1. Click the word for which you want to find an opposite or substitute.  2. Click the Review tab.  3. Click Thesaurus.  The Research task pane appears.  The word you selected appears here.  Click here to display a list of resources you can use to search for information.  A list of words with similar meanings appears. Each bold word represents a part of speech — a noun, a verb, and adjective — with a similar meaning to the word you selected.Each word listed below a bold word is a synonym for the bold word.  Antonyms are marked.  4. Point the mouse at the word you want to use in your document. Appears beside the word.  5. Click here to display a list of choices.  6. Click Insert.  Word replaces the word in your document with the one appearing in the Research task pane. |
| **T53** | 1. Click on Page Layout tab,  2. In the Themes group, click Themes.   1. Do one of the following:    - To apply a predefined document theme, under Built-In, click the document theme that you want to use.    - To apply a custom document theme, under Custom, click the document theme that you want to use.   NOTE: Custom is available only if you created one or more custom document themes. For more information about creating custom document themes, see [Customize a document theme](https://support.office.com/en-us/article/Apply-customize-and-save-a-document-theme-in-Word-or-Excel-da3f1e8e-2338-457c-977f-25f950016710#bm2) below.  3. If a theme that you want to use is not listed, click Browse for Themes to find it on your computer or network.  4. Click on line number arrow below mailing tab.  Downward menubar appears  5. select your desired line number. |
| **T54** | 1. Open the document you want to print preview.   To print only selected text, select that text.   1. Click the **File** tab.     The Backstage view appears.   1. Click **Print**.   A preview of your document appears here.   1. Click the arrows at the bottom to page through your document. 2. To magnify an area of a page, drag the Zoom slider.   6- Select a printer.   1. To print more than one copy, type the number of copies to print here. 2. Click the Settings button to print the entire document, text you selected, only the current page, or document elements such as document properties or a list of styles used in the document. 3. To print noncontiguous pages, type the pages you want to print, such as **1,5,6–9**, in the Pages box. 4. To print the document, click the **Print** button.   If you change your mind and do not want to print, click the **File** tab to return to the document window**.** |
| **T55** | 1- Click the File tab.  The Backstage view appears.  2- Click Protect Document.  3- Click Mark As Final.  A message explains that Word will mark the document as final and then saved.  4- Click OK.  Word saves the document and confirms that the document has been marked as final and editing commands are unavailable.  5- Click OK.  6- Click the File tab. |
| **T56** | 1. Click the file menu.  2. Click exit.  3. Warning will appear, Click on Save/Don’t Save.  Word will vanish. |
