# Supplementary material for: Touch or click friendly: Towards adaptive user interfaces for complex applications
Source: PLoS One. 2024 Feb 5;19(2):e0297056. doi: 10.1371/journal.pone.0297056 (PMC10843409; doi:10.1371/journal.pone.0297056)
Supplement: S3 Appendix — (DOCX) [file pone.0297056.s003.docx]

**Appendix B: QUESTIONAIRE**

**This is a general-purpose Questionnaire being used for academic purposes only. The information you provide will be strictly confidential. You are not required to give any personal details.**

1. My Gender is (Male / Female).

2. My age is (in years). a.18-25 b. 26- 33 c. 34-41 d. 42-49

3. My education is a. Undergraduate b. Graduate c. PhD

4. My subject area is a. Computer Science b. Information Technology c. Bioinformatics d.____

5. Handedness a. Right b. Left c. Ambidextrous (both)

6. I can comprehend the language (You can choose multiple options).

a. Urdu b. Punjabi c. Hindko d. English e.____

7. I can rate my vision as? a. Excellent b. Good c. Fair d. Poor

8. I have any color deficiency. a. Yes, b. No

9. I use it regularly (you can choose multiple options).

a. Microsoft Word b. Web browser c. E-mail d. games e. ___

10. I have one or more of the following touchscreen devices.

a. Smartphone b. Tablet c. laptop d. None

11. I have been using the touchscreen since

1. Never used it. b. Just started using it. c. Over six months. d. Over a year e. More than two years.

12. I have been using the laptop touchscreen since

1. Never using it. b. just started using it c. Over 6 months. d. Over a year e. More than 2 years

13. I have been using a computer/Laptop for

a. never used b. 0.5 to 1 year c. 2 to 3 years d. 4-5years’s e. more than 5 years

14. I have been using Microsoft Word for

a. never used b. 0.5 to 1-year c. 2 to 3 years d. 4-5 year’s e. more than 5 years

15. With which of the following input devices you are familiar (you can choose multiple options).

a. Mouse b. Touchscreen c. Touchpad d.___________

| **Modified SUS Questionnaire**  **Evaluate how much you agree with the statements given below with (Tick mark in appropriate column)**  **Strongly Disagree=1, Disagree = 2, Neutral = 3, Agree =4, Strongly Agree = 5.** | |
| --- | --- |
| \| **S. No** \| **Questions** \| **1** \| **2** \| **3** \| **4** \| **5** \| \| --- \| --- \| --- \| --- \| --- \| --- \| --- \| \| **1** \| When using Microsoft Word mouse is easy to use. \|  \|  \|  \|  \|  \| \| **2** \| I think that I would like to use the mouse frequently when using Microsoft Word. \|  \|  \|  \|  \|  \| \| **3** \| I found the mouse cumbersome to use when using Microsoft Word. \|  \|  \|  \|  \|  \| \| **4** \| Overall, I am satisfied with the mouse, when using Microsoft Word. \|  \|  \|  \|  \|  \| \| **5** \| When using Microsoft Word mouse helps me, be more productive. \|  \|  \|  \|  \|  \| \|  \|  \|  \|  \|  \|  \|  \| \| **1** \| When using Microsoft Word touchpad helps me, be more productive. \|  \|  \|  \|  \|  \| \| **2** \| Overall, I am satisfied with the touchpad, when using Microsoft Word. \|  \|  \|  \|  \|  \| \| **3** \| I found the touchpad cumbersome to use when using Microsoft Word. \|  \|  \|  \|  \|  \| \| **4** \| I think that I would like to use a touchpad frequently when using Microsoft Word. \|  \|  \|  \|  \|  \| \| **5** \| When using Microsoft Word touchpad is easy to use. \|  \|  \|  \|  \|  \| \|  \|  \|  \|  \|  \|  \|  \| \| **1** \| I think that I would like to use touchscreen frequently when using Microsoft Word. \|  \|  \|  \|  \|  \| \| **2** \| When using Microsoft Word touchscreen is easy to use. \|  \|  \|  \|  \|  \| \| **3** \| I found the touchscreen cumbersome to use when using Microsoft Word. \|  \|  \|  \|  \|  \| \| **4** \| When using Microsoft Word touchscreen helps me, be more productive. \|  \|  \|  \|  \|  \| \| **5** \| Overall, I am satisfied with the touchscreen, when using Microsoft Word. \|  \|  \|  \|  \|  \| |  |
