# Supplementary material for: Touch or click friendly: Towards adaptive user interfaces for complex applications
Source: PLoS One. 2024 Feb 5;19(2):e0297056. doi: 10.1371/journal.pone.0297056 (PMC10843409; doi:10.1371/journal.pone.0297056)
Supplement: S4 Appendix — (DOCX) [file pone.0297056.s004.docx]

**Appendix C: SCENARIO DOCUMENT**

CIIT’s History and Quick Facts

Historical Perspective

The Commission on Science and Technology for Sustainable Development in the South (COMSATS) is an international organization. It aims to reduce the ever-growing gap between the developed and developing world through useful applications of science and technology. The Third World Academy of Sciences (TWAS) initiated the proposal for the formation of COMSATS under the leadership of Nobel Laureate, Dr. Abdus Salam.

[2] The foundation conference of COMSATS was held in Islamabad on 4th & 5th October 1994. Representatives from thirty-six countries attended. The participants included twenty-two Ministers, members of the diplomatic community of Islamabad, and representatives of international organizations, like UNESCO, UNIDO, UNEP, and the World Bank.

[3] The conference decided that the Headquarters / Secretariat of the Commission would be based permanently in Islamabad, Pakistan, and the Head of State of Pakistan would act as the first Chairperson of the forum. It was agreed that the host -Government, Pakistan, would provide for the operational and administrative expenses of the Secretariat, while the development programs of the Commission would be supported and financed through Technical Assistance Fund. This fund would be established by contribution from member countries, income from services provided to member countries, grants from international agencies, and project funds if undertaken under the contract.

[4]The CIIT was established in 1998, as a project of the Commission on Science and Technology for Sustainable Development in the South (COMSATS), which is an inter-governmental organization with 21 member states in three continents: Asia, Africa, and Latin America, namely Bangladesh, China, Colombia, Egypt, Ghana, Iran, Jamaica, Jordan, Kazakhstan, Korea (DPRK), Nigeria, Pakistan, Philippines, Senegal, Sri Lanka, Sudan, Syria, Tanzania, Tunisia, Uganda, and Zimbabwe. Currently, CIIT has the status of a public sector degree-awarding higher education institution and was given a charter by the Federal Government in August 2000.

Established/Charter.

1998/August 2000

Vision

CIIT aspires to be both one of the top research institutions and one of the best higher education providers in the country. It envisages becoming a university by the name of “COMSATS University”, for which the legal documentation is under process with the Government of Pakistan. The vision being pursued by the CIIT is to become one of the top 100 universities in the developing world. The CIIT further intends to earn a place among the top 500 universities of the world by the year 2020.

Mission

The Institute's mission is threefold:

i) Research and Discovery

ii) Teaching and Learning

iii) Outreach and Public Service

Extend, apply, and exchange knowledge between the institute and society by applying scholarly expertise to intellectual, social, and technological problems, by helping organizations and individuals respond to their changing environments, and by making the knowledge and resources created and preserved at the institute accessible to the citizens. Using the resources of its multiple campuses in an integrated fashion, the Institute vies to strengthen the services to the state through the education of a modern workforce, research and development, technology commercialization, and partnership with business, government, and community groups.

Table 1

| Campuses | 08 (7 Campuses are ISO 9001:2008 Certified) |
| --- | --- |
| Faculties | 06 |
| Academic Departments | 18 |
| Research Centers | 10 |
| Total Library Books | 150,263 |
| Faculty | 3,118 including Research Associates |
| Distinction (go to) | 1,055 PhD Faculty and Academic Managers |
| Students | 35,971 as of Spring 2016 (including Virtual campus) |
