# Supplementary material for: Touch or click friendly: Towards adaptive user interfaces for complex applications
Source: PLoS One. 2024 Feb 5;19(2):e0297056. doi: 10.1371/journal.pone.0297056 (PMC10843409; doi:10.1371/journal.pone.0297056)
Supplement: S5 Appendix — (DOCX) [file pone.0297056.s005.docx]

Appendix D - Expertise Based Analysis

EFFECTIVENESS

The effectiveness was measured by using two metrics: task completion rate and the number of errors made while performing a task. The details are as follows.

**Task completion rate** is computed as the number of tasks completed successfully divided by the total number of tasks performed and multiplied by a hundred to get a percentage of the task completion rate. Figure 1 shows that the task completion rate with a mouse was higher as compared to a touchscreen or a touchpad in all three expertise groups. For the novices, the task completion rate was less while using a touchscreen as compared to a touchpad. This seems to be against the finding of the earlier research as discussed in section 2. The reason could be that for novice users the touchscreen was easiest to use, yet they made more errors with the touchscreen and spent more time correcting the errors. Thus, more tasks were left uncompleted as compared to a mouse or a touchpad. The explanation also correlates with literature as the literature suggests that touchscreen is not accurate and is more error prone [7].

Figure 1. Task completion rate in percentage by type of users while using the devices.

**The number of errors** is the errors made by participants while doing tasks with either of the input devices. Errors may be unintended actions, mistakes, and omissions users make while attempting tasks. For example, users make repeated attempts to perform some tasks and could miss a few steps while attempting the tasks.

Figure 2. Total number of errors made by the type of users while using the devices.

Figure 2 shows that participants made most errors with a touchscreen while the least errors made are with a mouse. Both mouse and touchpad are pointing devices having - pixel hot spots, leading to their better accuracy. It is noteworthy that while using a touchscreen, experts made the least number of errors while novices made the most errors. This also explains why novices have not completed most of the tasks while using a touchscreen. The results are aligned with the findings of the related literature.

EFFICIENCY

Efficiency is measured in terms of time used to complete a task. The efficiency can further be divided into two efficiency-based metrics: 1) Time-based efficiency and 2) Overall relative efficiency.

**Time-based efficiency** is referred to as time on a task. In other words, it measures the speed of work and is measured in goals/second units. Time-based efficiency is measured by using the following formula:

$\frac{\sum_{\mathbf{j=1}}^{\mathbf{U}} \sum_{\mathbf{i=1}}^{\mathbf{N}} \frac{\mathbf{Rij}}{\mathbf{Tij}}}{\mathbf{NR}}$ (1)

Where R is the number of users,

N is the total number of tasks (goals)

Rij is the result of task i by user j. If the task is completed Rij is 1/ not completed Rij is 0.

Tij is the time used by user j to complete task i

The x-axis of Figure 3 shows goals equated as tasks completed per second. The maximum value (0.09) in the graph shows a goal completed in approximately 11 seconds. As per data, expert and intermediate users performed more tasks per second with a mouse and least while using a touchpad. However, novice users performed more tasks per second with a touchscreen and least while using a touchpad. It shows that novice users are more comfortable with a touchscreen as it is a direct device, and no mapping/translation is required. One interesting point to note here is that intermediate skilled users performed better than experts while doing tasks with a mouse. This might be because expert users prefer executing tasks with a keyboard (shortcut keys) most often. However, due to the non-availability of the keyboard, their performance might have decreased with a mouse.

Figure 3. Efficiency in terms of goals/second

**Relative efficiency** is the ratio of the time taken by the users who completed the tasks divided by the total time taken by all the users. In other words, it is a measure of the efficiency with which users can perform their tasks on a given interface/system. A 100% efficiency means that all users can perform a task within the average minimum possible time required to complete that task. The relative efficiency is measured with the following equation.

$\frac{\sum_{\mathbf{j}=\mathbf{1}}^{\mathbf{U}} \sum_{\mathbf{i}=\mathbf{1}}^{\mathbf{N}} \mathbf{RijTij}}{\sum_{\boldsymbol{j}=\mathbf{1}}^{\boldsymbol{U}} \sum_{\boldsymbol{i}=\mathbf{1}}^{\boldsymbol{N}} \boldsymbol{Tij}}\times\mathbf{100}$ (2)

Figure 4 shows that the intermediate skilled and novice users were found relatively efficient or equal to the expert users in terms of touchpad usage. The percentage of performing tasks with a touchpad was low in all three groups. The main reason might be the fact that users do not use touchpads on their laptops as their main input device and connect a mouse instead. A touchpad is smaller in size and does not allow precise interaction. Control and display ratio 1:8 means that the motion of a finger on a touchpad is 1 unit then the motion of a pointer on the screen is 8 units which makes the use of the touchpad difficult.

Figure 4. The relative efficiency of the devices concerning the type of the users.
