# Supplementary material for: Touch or click friendly: Towards adaptive user interfaces for complex applications
Source: PLoS One. 2024 Feb 5;19(2):e0297056. doi: 10.1371/journal.pone.0297056 (PMC10843409; doi:10.1371/journal.pone.0297056)
Supplement: Attachment — Submitted filename: Comments for PONE_D_23_26336.docx [file pone.0297056.s009.docx]

Abstract

It is not clear how the recommendation in the last sentence follows from the objective of the study. The original question was about usability of the input device, but the recommendation is for a customized menu.

Introduction

First two paragraphs do not describe the purpose of the paper very well and lack direction. The introduction to the paper begins in earnest around line 43

Earlier in the introduction there needs to be some explanation of “task” and “action” based analysis and methods, and what this study is intending to do. As written, those terms are not specific.

The objective of this study is not made clear in the introduction. There is some potentially interesting exploration of existing research with the discussion around task classification, and the statements about challenges to this type of study are interesting. This needs further discussion in the introduction and a stronger, concise summary of how this study addresses the challenges. Some of this is detailed better in Section 2.

Section 2 – Related work

Lines 106-107 – The objective of the study should be clearly stated, and not need to be inferred. This sentence could clearly summarize the objective/aim of the study.

The term “performance” needs to be clearly defined, ideally in either the introduction or sections 2.1/2.2. The term “usability” also comes into section 2.2, and that is another that needs a quick definition. The measure is often time, or error frequency, but it’s not clear what’s being considered “good performance” in these tasks. Especially as the tasks are identified as “cut, copy, paste…” it’s unclear what metric would be “better” for these tasks. This is important since this is a comparison of devices, and presumably they all are capable of performing the task at some basic level.

Section 2.3 – the demographic comparisons of previous studies could be better communicated as a table, and of interest will be the task types and what the measure of “performance” was.

Section 2.4 – starts discussing some of the metrics of previous studies, which is good, although “comfort metrics” can be expanded upon. Of interest is whether these were entirely user reported, and whether that data was collected during or after the task. The statement in 169/170 is not supported by the preceding sentences, if it was a conclusion of the referenced study, that should be made more clear. The statement invites argument in this context, because this study is stated to be examining an application that employs a combination of tasks, and it sounds awkward to have to switch between input devices for related operations in a word processor.

Section 3 - Methods and Materials

The objective and applicability of this study is still not clear. The description of the tasks selected doesn’t seem to align to any use case or practical application; the tasks were selected based on the number of input operations and their placement on a very information-heavy tool ribbon. The Tool ribbon in MS 365 groups tasks/operations (roughly) according to the phase of document production/review…not ease of access for novice users. This is also the first place in the study that “novice users” is brought up at all, and the relevance of that classification to the study outcomes is not apparent. When selecting the optimal tool for anything, there is typically a distinct set of criteria for novice and expert users. If the measurement of accuracy and efficiency in task completion is the “performance” the study is seeking, novice users do not seem to be the ideal subjects.

Section 3.5 – 268-270 sentence does not make sense. I think the term you are looking for might be “heterogeneous” as homogeneous data sounds like it would be more readily generalizable. This sentence might not be very useful in detailing the method of analysis.

It is also unclear whether the demographic divisions were determined a priori, and if so, why. Why was five years considered to be a threshold of relevance?

280-282 “no uncorrected eyesight or color blindness” doesn’t make sense.

Section 4 – Experimental setup

317-318 it is unclear how a break prevents or minimizes boredom. If no other activities or movement were allowed, it seems more likely that this break might have impacted fatigue? Intuitively it seems that a simple break might actually increase boredom, in the absence of a directed task.

323-325 Who analyzed the videos and how was consistency/competence assured?

Section 4 – Results

Please address redundant section number

329 – if it is clear from research, please include a citation.

334-335 This is a lot of hypothetical and qualifying statements for a results section. It makes the communication unclear.

337-345 it is difficult to tell whether the ANOVA is appropriate in this case without a very clear statement of which variables are being analyzed and how those variables were being measured. The long quotation seems to suggest that a low level of significance was observed, but the authors do not state why that lower level is appropriate to this study.

Table 2 “Better” is a value judgement, a title that is more directly descriptive of what was measured in this study would be more clear in communicating results.

385-389 The description of variables here is good, but the opening statement, that this leads to “better customization” is very confusing and not linked to stated aims.

404-405 is troubling because it looks like the analysis method was selected

421-429 the specific variables and relationships need to be summarized more concisely, and without extensive qualifying statements somewhere. It remains unclear why this statistical analysis is relevant to any conclusion for the study.

There are interesting elements in this study and the data set and analysis can add to this field, but major revision to the presentation would help to frame the contribution and make the practical application for these findings more evident.
