# Supplementary material for: Touch or click friendly: Towards adaptive user interfaces for complex applications
Source: PLoS One. 2024 Feb 5;19(2):e0297056. doi: 10.1371/journal.pone.0297056 (PMC10843409; doi:10.1371/journal.pone.0297056)
Supplement: Attachment — Submitted filename: Response_PONE-D-23-26336_R1.docx [file pone.0297056.s011.docx]

The aims and relevance of the study are much more clearly described and supported in the revised manuscript. Thank you for the effort and thoughtful incorporation of previous comments.

The inclusion of clear definitions and the supporting standards/documents is much appreciated and significantly sharpens the impact of this paper in the body of related work. This is of particular value to readers in industry.

The methodology is better supported and contextualized in the “related work” section, thank you.

171-172 A sentence here describing how the method is similar, but the setting different would be beneficial. This is a minor revision, but would support the next sections very well. You have described the GOMS model nicely and the Appendix shows how you have applied it.

190 “Numerous research literature…” please include citations, or omit the sentence.

234—236 a random video of what? Just a random selection from the test set? Or videos of people doing similar tasks from another source?

Figure 1 contains a lot of excellent information, but some of it is a process flow, and some of it is supplemental information. Please rework to ensure the flow diagram summarizes sequential steps, and the supplemental information is either referenced or indicated separately.

The statistical analysis is much better described and much aided by the changes to the tables. Thank you.

273-275, Consider “fewer” instead of “lesser”…”lesser” indicates that the severity of an error was lower, whereas “fewer” means a lower total number of errors in execution.

323 “likeness” might be replaced by “users’ preference of a device”
