# Supplementary material for: Touch or click friendly: Towards adaptive user interfaces for complex applications
Source: PLoS One. 2024 Feb 5;19(2):e0297056. doi: 10.1371/journal.pone.0297056 (PMC10843409; doi:10.1371/journal.pone.0297056)
Supplement: Attachment — Submitted filename: Response to reviewers PONE-D-23-26336_R2.docx [file pone.0297056.s012.docx]

**Reviewer #1**

**Comment 1**: The aims and relevance of the study are much more clearly described and supported in the revised manuscript. Thank you for the effort and thoughtful incorporation of previous comments.

The inclusion of clear definitions and the supporting standards/documents is much appreciated and significantly sharpens the impact of this paper in the body of related work. This is of particular value to readers in industry.

The methodology is better supported and contextualized in the “related work” section, thank you.

**Response**: Thank you for your positive feedback! I'm pleased to hear that the revisions have clarified the aims and relevance of the study. I appreciate your recognition of the clear definitions and inclusion of supporting standards, which add value for readers in the industry. The improvements to the methodology's support and contextualization in the "related work" section are noted, and I'm grateful for your thoughtful comments. If you have any additional suggestions or areas for improvement, I'm open to further feedback.

**Comment 2**: 171-172 A sentence here describing how the method is similar, but the setting different would be beneficial. This is a minor revision but would support the next sections very well. You have described the GOMS model nicely and the Appendix shows how you have applied it.

**Response**:

Thank you very much for appreciating the text. We have now explained the settings that the existing literature used as well as made some changes in the text to align it well with the next sections. Please see lines 190-194.

**Comment 3**:

190 “Numerous research literature…” please include citations, or omit the sentence.

**Response:**

The sentence is now deleted**.**

**Comment 4**:

234—236 a random video of what? Just a random selection from the test set? Or videos of people doing similar tasks from another source?

**Response**:

We have changed the text to “Another author of the study validated the extracted information by choosing and watching random 10 videos from the recorded videos of participants doing tasks in the experiment and matching the data with the Excel sheet data.” Please see lines 255 – 257.

**Comment 5**:

Figure 1 contains a lot of excellent information, but some of it is a process flow, and some of it is supplemental information. Please rework to ensure the flow diagram summarizes sequential steps, and the supplemental information is either referenced or indicated separately.

**Response**:

We have now formulated a new Figure 1. We hope that his figure is a true representation of the experimental setup.

**Comment 6**:

The statistical analysis is much better described and much aided by the changes to the tables. Thank you.

273-275, Consider “fewer” instead of “lesser”…”lesser” indicates that the severity of an error was lower, whereas “fewer” means a lower total number of errors in execution.

323 “likeness” might be replaced by “users’ preference of a device”

**Response**:

We are very thankful for the encouragement and appreciation. The word “fewer” is now used as indicated by the reviewer in the entire section 5.2 i.e., “effectiveness”. The word “likeness” is replaced by "preference”.

**Reviewer #2**:

The authors have significantly improved the manuscript. However, the following comments need to be addressed before it can be accepted for possible publication.
**Comment 1:**

**Related Work**
The insights/conclusions that can be derived from the existing literature are still missing and need to be included. Currently, these are missing. For instance, information like which input device is better for what type of tasks? It would be beneficial to the reader if a summary of the same is also included in Table 1.
The authors seem to have misinterpreted the reviewer's previous comment on this which read: “The way the existing literature has been reported in Related Work needs to be improved. It would be helpful if conclusions/insights derived from those studies are reported (briefly) so that the reader benefits from reading it. Just reporting the number/gender of participants, number of tasks, and what has not been done in the previous studies is not very helpful.” The reviewer meant that the findings/conclusions from the previous studies should at least be included briefly.
**Response**:

We are sorry for the misinterpretation. We have tried to provide insight/conclusions as per the reviewer's recommendation. We have added three more columns in Table 1 and the devices that are better for the type of tasks in the text. We hope this change will improve the related work section. Please see lines 111 – 128 as well as the modified Table 1.

**Comment 2**:

Experimental Setup

Fig. 1 needs to be improved significantly so that the experimental methodology is clear from the start to the data analysis. There is a lot of unnecessary information in Fig. 1, such as scenario text copied to the desktop, and participants invited to college. Make it crisp. The current figure is nothing more than a summary of text that is already there in Section 4.
**Response**:

We have now formulated a new Figure 1. We hope that his figure is a true representation of the experimental setup.

**Comment 3**:
Section 5.1
“As Table 3 shows, out of fifty-six cases, 27 or 48% of the tasks’ execution time was less with a mouse, while 21 (37%) tasks were executed with significantly less execution time. Furthermore, 3 tasks had a large effect size (η2 ≥ 0.14), 8 tasks had a medium effect size (η2 ≥ 0.06), and 10 tasks had a small Effect size (η2 ≥ 0.02).” Please write explicitly whether these 21 tasks are out of the 27 tasks mentioned earlier. Also, what do the effect sizes signify here need to be stated.
**Response**:
We have mentioned that 21 tasks were out of the 27 tasks in the following words: “*Out of these 27 tasks,* 21 (37%) tasks were executed with significantly less execution time”.

Though the effect size shows the magnitude or size of the observed relationship, the effect sizes in this study are reported for information purposes only and as per the journal reporting guidelines. For the customization of the menu, we need to group as many tasks as possible into either touch or click-friendly. Therefore, the grouping is discussed in **lines 326 – 333** where it is also discussed that the tasks above 0.01 or 0.05 level and below 0.3 threshold are added in the customized menu. Even the tasks, above 0.3 level could be adjusted in the customized menu depending on their inclination toward touch or click friendliness.

**Comment 4**:

Section 5.3
What is the rationale for choosing Efficiency with effectiveness as a covariate and not the other way around i.e., Effectiveness with efficiency as a covariate?
While Table 6 now reports the results in percentages, the text still contains numbers. Reporting of percentages along with numbers in the main text will make it more readable.

**Response**:

The basic objective of grouping the tasks in a customizable menu can be achieved via either of the analyses i.e., choosing efficiency with effectiveness as a covariate or the other way around. However, for effectiveness, as can be seen from Table 6, have very limited tasks inclined towards a touchscreen and are highly inclined towards click friendliness. Both a mouse and a touchpad are click friendly, therefore, variation of customization can be better achieved from the former analysis and thus was conducted. The fact is mentioned in lines 384 – 391.

For Table 6, percentages are now also represented along with numbers. Please see lines 317-320.
